# A Physiologically-Based Pharmacokinetic Modeling Approach Using Biomonitoring Data in Order to Assess the Contribution of Drinking Water for the Achievement of an Optimal Fluoride Dose for Dental Health in Children

**DOI:** 10.3390/ijerph15071358

**Published:** 2018-06-28

**Authors:** Keven J. Jean, Nancy Wassef, Fabien Gagnon, Mathieu Valcke

**Affiliations:** 1 Institut National de Santé Publique du Québec (INSPQ), Montréal, QC H2P 1E2, Canada; keven.biomed@outlook.com (K.J.J.); nancy.wassef@inspq.qc.ca (N.W.); fabien.gagnon@inspq.qc.ca (F.G.); 2 Département de Santé Environnementale et Santé au Travail, École de Santé Publique de l’Université de Montréal (ESPUM), Montréal, QC H3C 3J7, Canada; 3 Centre de Recherche du Centre Hospitalier Universitaire de Sherbrooke (CRCHUS), Sherbrooke, QC J1H 5N4, Canada

**Keywords:** biomonitoring, dental health, drinking water, fluoride, pharmacokinetic modeling

## Abstract

Due to an optimal fluoride concentration in drinking water advised for caries prevention purposes, the population is now exposed to multiple sources of fluoride. The availability of population biomonitoring data currently allow us to evaluate the magnitude of this exposure. The objective of this work was, therefore, to use such data in order to estimate whether community water fluoridation still represents a significant contribution toward achieving a suggested daily optimal fluoride (external) intake of 0.05 mg/kg/day. Therefore, a physiologically-based pharmacokinetic model for fluoride published in the literature was used and adapted in Excel for a typical 4-year-old and 8-year-old child. Biomonitoring data from the Canadian Health Measures Survey among people living in provinces with very different drinking water fluoridation coverage (Quebec, 2.5%; Ontario, 70% of the population) were analyzed using this adapted model. Absorbed doses for the 4-year-old and 8-year-old children were, respectively, 0.03 mg/kg/day and 0.02 mg/kg/day in Quebec and of 0.06 mg/kg/day and 0.05 mg/kg/day in Ontario. These results show that community water fluoridation contributes to increased fluoride intake among children, which leads to reaching, and in some cases even exceeding, the suggested optimal absorbed dose of 0.04 mg/kg/day, which corresponds to the suggested optimal fluoride intake mentioned above. In conclusion, this study constitutes an incentive to further explore the multiple sources of fluoride intake and suggests that a new balance between them including drinking water should be examined in accordance with the age-related physiological differences that influence fluoride metabolism.

## 1. Introduction

Intentional fluoridation of drinking water has been in use as early as the 1950s in order to prevent tooth decay both in children and adults. Based on data extracted from McClure’s [1] work, for the past several decades, an intake (that is, an external dose) of 0.05 mg/kg/day of fluoride has been considered to correspond to an “optimal fluoride intake” for caries prevention while minimizing fluorosis risk. It is the underlying population intake target behind the artificial fluoridation of drinking water at a concentration varying between 0.5 mg/L and 1 mg/L. This is now generally considered by many dental health professionals as a key public dental health measure [2]. Such fluoridation is recommended by the World Health Organization and supported by several organizations such as Health Canada, the Canadian Dental Association, the Centers for Disease Control and Prevention (CDC), and the United States Environmental Protection Agency (US EPA) [3]. Fluoride’s mechanism of action to prevent caries relies on its effect on tooth enamel, which is mainly composed of hydroxyapatite crystals. Exposure to fluoride results in the substitution of the hydroxy group by the fluoride anion to produce fluoroapatite. At the same time, hydroxyapatite is soluble at a pH of 5.5 and fluoroapatite is soluble at pH 4.5. Lactic acid produced by cariogenic bacteria can cause the pH at the surface of enamel to drop to between 5.5 and 4.5, which causes demineralization of the enamel. Therefore, the caries preventive effect of fluoride stems from its ability to be incorporated into dental enamel and form fluoroapatite, which is resistant to demineralization at a lower pH [4].

In Canada, the proportion of the population with access to intentionally fluoridated water is currently about 37% [3], but interprovincial disparities on this proportion, which do not vary throughout the year as a function of the season (contrary to some other places in the world), is important. Furthermore, approximately 70% of Ontario’s population now benefit from this public health measure. The public health concern has decreased from 12% in the early 2000s to less than 2.5% today in the neighboring Province of Quebec [5]. In this context, it is interesting to note that, according to the Ontario Association of Public Health Dentistry (OAPHD), for the 2012–2013 school year, Grade 2 students in Ontario had a mean DMFT index (decayed, missing, or filled teeth due to caries) in primary and permanent teeth (dmft+DMFT) of 2.22 with 50% of students being caries free. This result is based on screening data voluntarily reported by participating public health units to the OAPHD [6]. In Quebec, the Clinical Study on the Oral Health of Quebec Elementary School Students in 2012–2013 revealed, for the same age group, a mean dmft+DMFT of 2.67 with 44% of students being caries-free [7]. It should be recalled that dental caries have a multifactorial etiology and that the study groups were neither sampled nor weighted, according to fluoride exposure.

Proponents of community water fluoridation (CWF) highlight its contribution to address social health disparities [8]. In addition, nearly one in three Canadians does not have dental insurance and cannot receive the treatments needed due to their cost [9]. In Canada, dental caries treatments are second only to mental health problems with regard to societal costs ever since the mid-1970s [10]. Since tooth decay can affect children and adults as well as the elderly, the protective effect of fluoridated water consumption benefits everyone regardless of age. However, overexposure to fluoride during the tooth development can cause dental fluorosis. Mild to severe dental fluorosis is observed more frequently when the concentrations of fluoride in drinking water increase. Chronic exposure to high levels of fluoride may also cause more severe effects such as skeletal fluorosis and bone fractures [3,11,12,13,14], but the relevant studies have many limitations inherent to their ecological design. In addition, Health Canada’s fluoride expert panel concluded a decade ago that the scientific studies available at the time did not support a link between intentional fluoridation of drinking water and cancer or any other adverse effect than fluorosis [15]. Since that time, further studies have raised some concerns regarding the neurodevelopmental toxicity of fluoride. However, at exposure levels that are generally well above the exposure levels correspond to intentional fluoridation of drinking water [16]. Additional studies have further supported the presumption that drinking water fluoridation is not associated with an increased risk of osteosarcoma [17,18,19,20,21] nor with altered thyroid function [22].

In contrast with the era when the optimal fluoride concentration in drinking water was determined, the population is now exposed to multiple sources of fluoride that contribute to the total daily fluoride intake (TDFI) such as through the use of fluoride toothpaste but also by ingesting a greater variety of foods and beverages as well as existing exposure routes such as soil ingestion and inhalation of residual air concentrations. Therefore, it appears relevant to re-examine the contribution of drinking water fluoride for caries prevention purposes today. The availability of both a physiologically-based pharmacokinetic (PBPK) model for fluoride [23] as well as population biomonitoring data for this element within the Canadian Health Measures Survey (CHMS) [24], which is combined with the acknowledged disparity between drinking water fluoridation coverage between two provinces mentioned above, represent a unique opportunity in this regard. The considerable statistical weight of the participants from the provinces of Quebec and Ontario within the whole CHMS allows the extraction of data specific to these provinces and considered as reasonably representative of their population’s exposure to the measured chemicals including fluoride [25].

The objective of this work was, therefore, to apply a PBPK modeling approach based on fluoride biomonitoring data from two CHMS provinces with very different drinking water fluoridation coverage in order to examine the resulting contribution of drinking water to achieving an optimal dose of fluoride in children from a caries prevention perspective.

## 2. Materials and Methods

The approach followed here relies on the use of a PBPK model in order to estimate internal exposure in the provinces of Quebec and Ontario using corresponding fluoride biomonitoring data from the second and third cycles (2009–2013) of the CHMS. The obtained doses are then compared to the suggested optimal intake for dental health of 0.05 mg/kg/day. Lastly, the internal dose metrics (IDM) in blood and urine corresponding to the chronic consumption of 0.7 mg/L fluoridated water were compared to the same IDM associated with the suggested optimal intake of 0.05 mg/kg/day.

### 2.1. PBPK Model Development and Validation

#### 2.1.1. Model Structure

**Figure 1:**
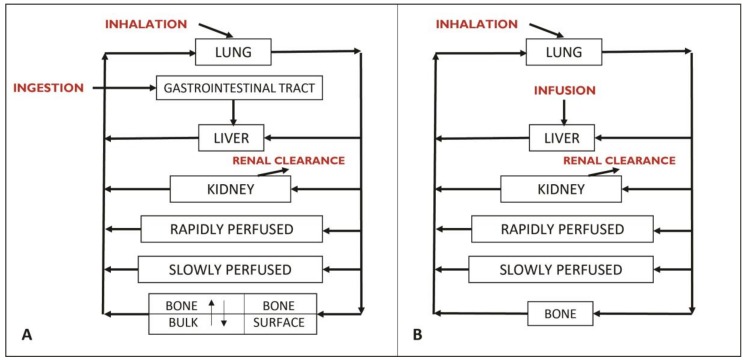
Conceptual diagram of (**A**), the original PBPK model of Rao et al. [23] and (**B**) the PBPK model simplified for the purposes of this work. Each box represents a compartment (an organ or a set of organs). The arrows symbolize the distribution of fluoride through the bloodstream.

The PBPK modeling approach used in this work is based on the model developed by Rao et al. [23] to simulate the bone kinetics of fluorides for life-long chronic exposure in rats and humans (Figure 1).

The initial model of Rao et al. [23] considers the subdivision of the bone compartment into the surface bone and the inner bulk bone in order to simulate the bone kinetics of fluoride in detail. The original objective of these authors was to model the variation of the bone fluoride concentration for a period of several years. For the purposes of the current work, however, solely the blood and urinary concentrations of fluoride were of interest. In addition, since the model aimed to simulate fluoride’s kinetics in children, the targeted population in the current study, in whom there is virtually no bone resorption because of growth, the inclusion of a compartment to consider the mobilization of bone fluoride and its release into the blood circulation was not required. Therefore, the original Rao et al. [23] model was simplified by considering a single bone compartment, which was reproduced in Microsoft Excel™ by Haddad et al. [26] and modified to specifically reflect the kinetics of fluoride in children.

#### 2.1.2. Model Parameters

In order to adapt the model of Rao et al. [23] in children, the fixed values of the physiological parameters used in the original model were replaced by formulas described in the literature [27,28,29,30]. These equations allow for accounting the age-specific and body weight-specific variations of blood flow and volume in the child. In doing so, average human equations for both sexes were used because provincial biomonitoring data for the current work were not distinguished by gender. The tissue partition coefficients of the Rao et al. [23] model were applied unchanged. The model considers that fluorides are solely eliminated by renal clearance. Therefore, the plasma clearance formula used by Rao et al. [23] was normalized to body weight to the 0.75th power. In a longitudinal dog study, bone clearance and renal clearance accounted respectively for 90% and 10% of plasma clearance in the pup and 50% each in the mature dog [23]. Assuming this relation is linear, this reasoning was used to draw two lines (0, 90, 18, 50) and (0, 10, 18, 50) whose slopes make it possible to obtain the percentages of the plasma clearance attributable to bone clearance or renal clearance as a function of age. By multiplying these percentages by plasma clearance, values for bone clearance and renal clearance were obtained.

Since the model aims to consider various sources of fluoride exposure such as air, tap water, soil (dust), fluoride toothpaste, food, and beverages (bottled water included), source-specific bioavailability factors were assigned in the model according to available literature [11]. Bioavailability factors of 83%, 100%, 100%, and 40% were used for water [31,32,33], air [34], fluoride toothpaste [35], and diet [36], respectively. For soil, the same bioavailability factor used for diet was attributed given that the factors influencing its bioavailability are similar. Due to the lack of available data on oral absorption constants from the GI tract in human PBPK models for fluoride, direct infusion in the liver was assumed, which triggers the withdrawal of the GI track compartment from the initial model (Figure 1B). Therefore, any daily dose ingested would be transformed into a minute-based infused dose in the liver over 24 h.

#### 2.1.3. Model Validation

For the purpose of model validation, data described in several published studies [37,38,39,40,41] in which fluoride intake was estimated and its urinary excretion measured were used in order to compare model simulations with observed data for given exposure conditions of specific child subjects. Additionally, among the available studies with measured fluoride intake and excretion in 24-h urine samples, the need for study participants to be aged eight years and under, in accordance with the targeted population for the modeling, was retained as a selection criterion. Since no such studies were found for children in Canada, studies from various countries (Venezuela, Chile, Germany, and United Kingdom) were selected to validate the model. Table 1 shows the five studies selected for fluoride intake and the amount of excreted urinary fluoride they measured.

### 2.2. Exposure Scenarios

#### 2.2.1. Subjects of Interest

Since this study aimed to interpret and use Canadian biomonitoring measures of fluorides, specific PBPK models for children of the corresponding age were built. In doing so, the median age of the two CHMS child age groups, was entered in the generic model described in Section 2.1. Specifically, distinct models for 4-year-old and 8-year-old children were built. Additionally, the 3-year to 5-year age group (median age: 4 years) was selected to evaluate the risk of dental fluorosis. In this age group, permanent teeth are still in development. Although the esthetic effects of dental fluorosis on permanent anterior teeth are generally considered to have taken place from birth to age 3, there was a lack of studies permitting a suitable validation of the model for this group. Complimentarily, the 6-year to 11-year age group (median age: 8 years) was selected in order to bring out insights on the possible age-related physiological differences that influence fluoride metabolism. The 50th percentile values of the World Health Organization (WHO) growth charts adapted for Canada in 2014 [42] were used to determine body weights and heights of the targeted median children. Then, data reflecting the Canadian exposure context were sought. The intake considered for fluoride toothpaste originated from an internal consultant report discovered by Health Canada based on data collected in the early 2000s concerning toothpaste containing between 996 and 1455 ppm of fluoride [11] while air and soil intakes were taken from the INSPQ [43]. Data on food and beverage intake from a survey of fluoride intake in the Canadian diet were also used [11]. With respect to the daily rate of water consumption, direct water consumption from a UK study [44] was selected. This was due to available Canadian data that did not concern the specific ages targeted herein or include indirect water consumption already taken into account by the food and beverage intake. Age-specific parameter values are presented in Table 2.

#### 2.2.2. Model Simulations of Internal Dose Metrics of Interest

**Figure 2:**
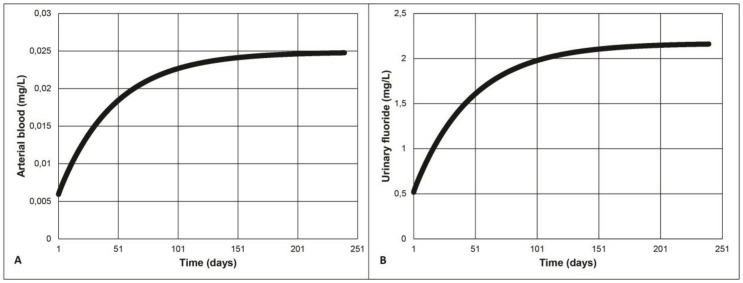
(**A**) Arterial and (**B**) urinary fluoride concentration in a 4-year-old child continuously exposed to 0.7 mg/L of fluoridated water.

Continuous fluoride exposure until the steady-state is reached, after approximately 150 days for both arterial blood and urinary fluoride (see example in Figure 2), was simulated with the models. Given that CHMS biomonitoring data, which will be used further, result from spot urine samples, an assumption that the measured urinary fluoride is a consequence of steady-state exposure is made. This presumption is a state-of-the art hypothesis for the interpretation. For instance, Biomonitoring Equivalents (BE) [45] of data was collected in large biomonitoring surveys such as the National Health and Nutrition Examination Survey (NHANES) [46], the German chemical Exposure Study (GERes) [47,48], and CHMS [49] with model-derived or model-simulated exposures.

By entering the available fluoride’s external exposure data for Quebec and Ontario as input in the PBPK model, the resulting urinary levels can be compared to the corresponding CHMS values, which exhibit significantly greater population mean urinary fluoride concentration in Ontario as compared to Quebec [25]. Specifically, a 24-h steady state amount of excreted urinary fluoride was obtained by subtracting the simulated amount of urinary fluoride excreted after 149 days of continuous exposure from the same amount excreted after 150 days.

#### 2.2.3. Exposure Scenarios Simulated

**Figure 3:**
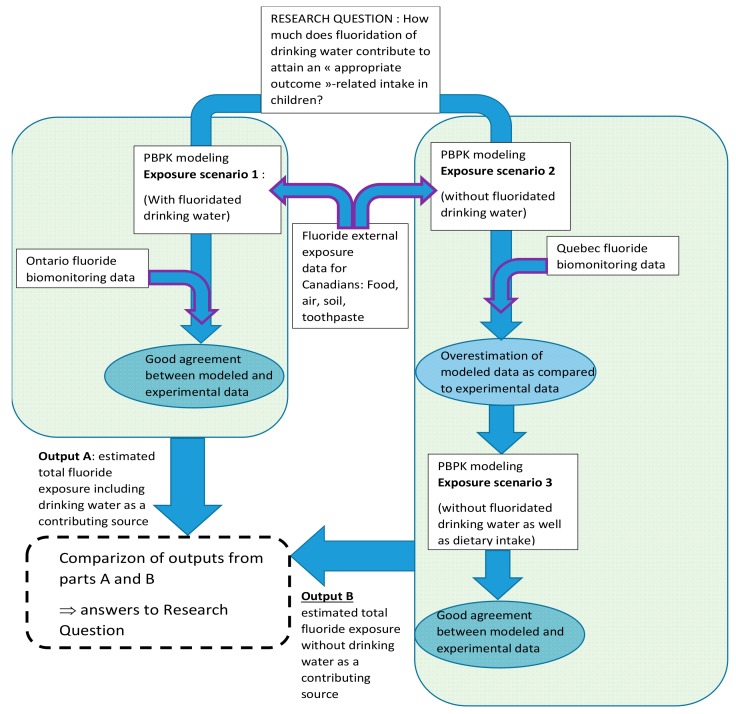
Flow chart describing the methodological rationale underlying the fluoride exposure scenarios simulated by PBPK modeling in order to examine the original research question.

Three exposure scenarios were considered. In the first scenario, exposure for a 4-year old and an 8-year old child from Ontario is modeled based on intakes listed in Table 2 from air, soil, diet, fluoride toothpaste, and fluoridated water (Figure 3). Since 70% of Ontario’s population including children has access to fluoridated water at 0.7 mg/L. An equivalent average fluoride concentration of 0.5 mg/L was calculated assuming that the remaining 30% non-fluoridated water had a concentration of 0.05 mg/L (detection limit). In the second scenario, in the deemed representative of a 4-year old or an 8-year old child in Quebec, it is assumed that the only difference in exposure sources lies in the much lower access to fluoridated water at 0.7 mg/L. Therefore, this second scenario was modeled with 2.5% of the Quebec child population consuming fluoridated water at 0.7 mg/L for an equivalent average of 0.06 mg/L (if 97.5% of inhabitants drink non-fluoridated water at a concentration of 0.05 mg/L).

As shown later (see Results), the simulation of the second scenario tends to overestimate the urinary concentration of fluoride. In order to evaluate the contribution of drinking water to TDFI in Quebec, the consistency between input parameters used in the model and reality, presumably reflected accurately by CHMS data, is required. This contribution was evaluated differentially by modeling the daily amounts of urinary fluoride excreted at steady-state levels assuming the exposure conditions present in Ontario vs. Quebec and comparing them to those modeled for the TDFI using available fluoride intake input parameters. Therefore, for determining the contribution of drinking water to the overall fluoride exposure, it was necessary to account for the fact that fluoride concentrations in water may affect those found in foods via cooking and preparation of processed food. Therefore, the third scenario was modeled without fluoride intake from the diet to better reflect the exposure of a 4-year old or an 8-year old child in Quebec.

### 2.3. Determination of Mean Absorbed Fluoride Dose in Average Children from Quebec and Ontario Using Biomonitoring Data

Since the present work relies in part on biomonitoring data, an absorbed dose is deemed more relevant to work for the purpose of the present study than an intake. Therefore, the calculated total ingested fluoride intake from water and diet were converted into corresponding absorbed doses by applying the relevant bioavailability factors of 83% and 40% presented above (see Appendix A Table A1). A mean resulting absorbed dose across all ages investigated (1–12 years old) of 0.04 mg/kg/day was obtained and was, henceforth, used for the purpose of this work.

Based on the mean and 95th percentile values of urinary concentrations of fluoride measured in the two CHMS provinces, corresponding absorbed doses in Quebec and Ontario could be modeled using the solver complement in Microsoft Excel™. The solver provides a value for a given parameter by modifying one or more model parameters while including constraints as needed. Therefore, CHMS urinary concentrations were converted to excreted amounts of urinary fluoride using daily urinary fluoride excretion rates [50]. The converted CHMS fluoride amount was entered into the solver as a 24-h steady state parameter and the model was only allowed to change the absorbed TDFI parameter while retaining the values and equations for all other parameters. The only constraint in this case was that the TDFI had to be a positive number (greater than zero). The solver then made it possible to determine the absorbed dose associated with the amount of urinary fluoride. The absorbed doses from Quebec and Ontario obtained by using the solver with CHMS urinary fluoride amounts at the mean and 95th percentile values could then be analyzed to determine if (1) the absorbed dose of 0.04 mg/kg/day detailed above was reached in those provinces and (2) the resulting intakes correspond to the total intakes computed in Section 2.2.2 considering different exposure levels via drinking water fluoridation.

### 2.4. PBPK Model Sensitivity Analyses

In order to know the impact of the variation of each parameter on the predicted internal concentrations of fluoride, a sensitivity analysis of the model parameters was carried out, according to the following formula (Valcke & Krishnan [29]).

(1) $${\mathrm{SI}}_{P}=\frac{{\mathrm{Aexc}}_{F_{2}}-{\mathrm{Aexc}}_{F_{i}}}{P_{2}-P_{i}}\times \frac{P_{i}}{{\mathrm{Aexc}}_{F\_i}}$$

where SI_P is the sensitivity index of the parameter “P”, Aexc_F_2_ is the quantity of urinary fluoride excreted over 24 h at a steady-state after reduction of the “P” parameter by 2% of its initial value, Aexc_F_i_ is the initial value of the quantity of urinary fluoride excreted over 24 h at a steady-state, P_i_ is the initial value of a parameter “P”, and P_2_ is its value after being reduced by 2% of its initial value.

## 3. Results

### 3.1. Model Validation

**Figure 4:**
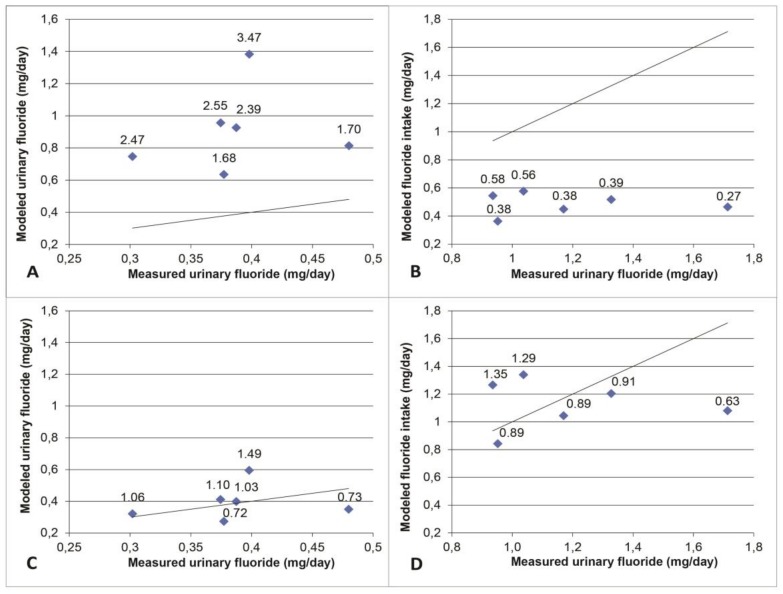
Results of model simulation of exposure data in the literature as a function of experimental biological measures in children aged 4 to 7 years. Each point represents a study while the line represents a perfect agreement. The values at the top of the points represent the ratios between the modeled values and the experimental values. Panels (**A**,**B**) illustrate, respectively, the over-estimation and underestimation of the modeled values. Panels (**C**,**D**) show the predicted values around the perfect agreement line when adjusted by multiplying by the adjustment factor of 0.43 in (**C**) and 1/0.43 in (**D**).

For intakes ranging from 0.04 mg/kg/day to 0.08 mg/kg/day in the studies used as validation, the amounts of urinary fluoride predicted by the PBPK model were always greater than the amounts measured (Figure 4). In order to overcome this systematic overestimation by the model, the average ratios “urinary fluoride measured/urinary fluoride modeled” were calculated to obtain the average empirical adjustment factor of 0.43. This factor was incorporated into the final model prior to running the simulation of exposure scenarios and model-based interpretation of biomonitoring data for which the results are presented hereafter in Section 3.2 and Section 3.3.

### 3.2. Modeled Exposure Scenarios

Table 3 shows that the modeled urinary concentrations of fluoride for the first scenario are slightly higher than the concentrations measured in Ontario’s CHMS participants with ratios of 1.02 and 1.09. For the second scenario, the modeled concentrations were much higher than those measured in Quebec’s CHMS participants with ratios of 1.81 and 1.78. This suggests that the modeled exposure for this scenario does not correspond to the actual exposure likely due to one or more sources of fluoride are overestimated for Quebec. The concentrations modeled for exposure in the third scenario approximate the measured concentrations while remaining higher with ratios of 1.47 and 1.41.

Table 4 shows the average absorbed fluoride doses, which were modeled based on CHMS urine concentrations using Microsoft Excel™’s solver complement. In Quebec, it is lower than the suggested optimal absorbed dose of 0.04 mg/kg/day. In Ontario, this dose is reached and even exceeded. However, when looking at the 95th percentile, the suggested optimal absorbed dose is exceeded in both provinces.

### 3.3. Contribution of Drinking Water to the Total Intake of Fluoride

**Figure 5:**
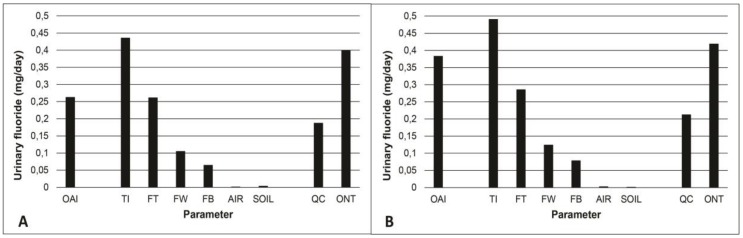
Modelled excreted fluoride over 24 h at a steady-state, according to the different intakes considered in the 4-year-old (**A**) and 8-year-old (**B**) child. The data from Table 2 were used for fluoride sources. These modeled data are compared to CHMS biomonitoring geometric mean data for cycles 2 and 3 combined for Quebec (QC) and Ontario (ONT) for children aged 3–5 years-old (**A**) and 6–11 years-old **(B)**. Symbols: OAI: suggested Optimal absorbed intake of 0.04 mg/kg/day. TI: Total (absorbed) intake. FT: Fluoride toothpaste. FW: 0.07 mg/L fluoridated water. FB: Food and beverages.

Figure 5 shows the contribution, based on PBPK model simulations, of different fluoride sources (see Table 2) to total intake as well as amounts or urinary fluoride for Quebec and Ontario converted from CHMS biomonitoring data. The contribution of air and soil is minimal. The most important source is toothpaste followed by fluoridated water at 0.7 mg/L and diet. The TDFI when the water is fluoridated at 0.7 mg/L is above the suggested optimal intake, which is slightly higher than what was measured in Ontario. In Quebec, where the majority of fluoride exposure is through ingestion of fluoride toothpaste, the intake measured by the CHMS is even lower than the modeled intake from fluoride toothpaste.

### 3.4. Sensitivity Analyses

**Figure 6:**
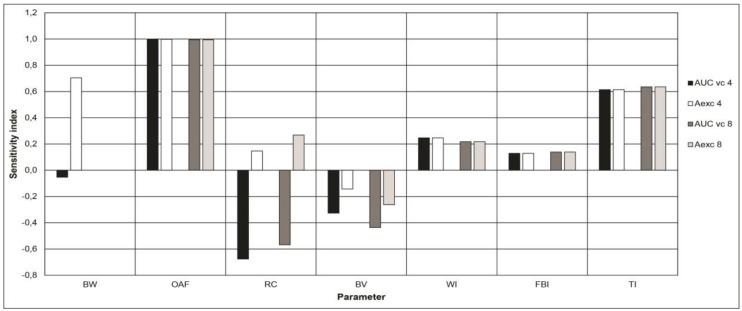
Sensitivity indices of the model parameters above the arbitrarily determined threshold of 0.05. Symbols: BW: Body weight. OAF: Oral absorption fraction. RC: Renal clearance. BV: Bone volume. WI: water intake. FBI: Food and beverages intake. TI: Toothpaste intake. AUC vc 4: Area under the curve of venous concentrations of fluorides over 24 h for a 4-year-old child. AUC vc 8: Area under the curve of venous concentrations of fluorides over 24 h for an 8-year-old child. Aexc 4: Amount of urinary fluorides excreted over 24 h for a 4-year-old child. Aexc 8: Amount of urinary fluorides excreted over 24 h for an 8-year-old child.

Figure 6 shows the parameters with the highest sensitivity indexes (SI) in the model. These include body weight, oral absorption fraction, renal clearance, bone volume, and fluoride intake (by using water, food, and toothpaste ingestion). For concision reasons, Figure 6 only includes parameters exhibiting an SI equal to or greater than 0.05.

## 4. Discussion

To the best of the author’s knowledge, this study is the first of its kind considering a coupled PBPK/biomonitoring data modeling approach to the comparison of fluoride intake in children living in regions with significantly differences in their access to intentionally fluoridated drinking water. PBPK modeling is an extensively used approach in order to interpret population biomonitoring data on chemical exposures [51,52,53,54,55,56,57,58], but it has never been applied to fluoride in this perspective nor has it been applied to fluoride exposure through drinking water from a dental health perspective. Therefore, the comparison made regarding the fluoride intake in children from two Canadian provinces differing in their access to optimally fluoridated water proposes new methodological insights with regard to future fluoride intake assessments.

This study suggests that drinking water fluoride still represents an important contribution for children under 8 years of age to attain the suggested daily optimal fluoride intake to prevent caries, but that a reduction in the intake of fluoride could also be desirable from the perspective of the most exposed children in order to prevent them from exhibiting a total exposure that may exceed toxicological reference values. Such a reduction could still be made while continuing to benefit from the protective effects of fluoride against tooth decay.

Although fluoride is classified as a non-essential mineral, it does have a protective effect against tooth decay. As any other preventive measure, its relevance is directly related to the risk of developing caries. It may not be relevant to use fluorides in populations who are not at risk of developing dental caries. In addition, fluoride’s effect being predominantly topical, it is important not to confuse the systemic absorption of fluoride with its topical effect since “topical fluorides” will always have some degree of ingestion and “systemic fluorides” will exert topical effects through saliva and crevicular fluid [59].

The use of an “optimal fluoride dose” based on McClure’s work, though controversial, remains the most appropriate population-level estimate for the purposes of this study because there is no other estimate currently available to predict how much fluoride is likely to confer a caries preventive effect while minimizing the risk of developing fluorosis. Although the relevancy of the notion of “optimal fluoride dose” to prevent dental caries exceeds the scope of this paper, it is worth mentioning that it has recently been suggested that the term optimal or “optimum” be dropped in favor of defining a value that provides “appropriate outcomes” for both caries and fluorosis [60]. However, considering the varying levels of caries risk between population subgroups as well as physiological differences affecting fluoride metabolism and absorption, a range of values may be more appropriate for future population level modeling studies [61].

By using the daily urinary fluoride presented in Figure 5, the contribution of drinking water fluoridated at 0.7 mg/L was calculated as 25% of the TDFI. At first glance, it may seem different from what other studies have found. In a US EPA relative source contribution analysis based on a mean exposure scenario in the United States, a relative contribution of 42% was found for children between 4 and 6 years old [62]. However, the fluoride concentration used in their scenario was 0.87 mg/L and the 90th percentile of water consumption (0.943 L/day) was used rather than the mean (0.442 L/day), which can partly explain the difference with the present analysis. Health Canada’s assessment [11] based on exposure scenarios using daily Canadian intakes suggests a contribution of water to the TDFI of 48%. Both direct and indirect water consumption were included in that assessment. If only direct water consumption was considered, as is the case in the present study, drinking water contribution would drop to 17%. In another study [63], using a three-day food diary and samples collected from various fluoride sources (tap water, drinks, foods, toothpastes, and tooth-brushing expectorate) consumed by 3–4 year old children residing in the Gaza Strip, water contribution to the TDFI was found to be 12%. The authors of that study mention that very little tap water was used as a drink. Therefore, the lower contribution of water to the TDFI. Estimates of fluoride intake by analysis of various sources with a fluoride ion selective electrode were made in South India in order to assess drinking water contribution to the TDFI, which was evaluated at 39% in 3 year old to 10 year old children [64]. On the other hand, fluoride toothpaste was not included in that assessment nor mentioned in the article. If the fluoride toothpaste intake considered in the current study was added in their contribution assessment, a relative water contribution to the TDFI value of 25% can be obtained. Therefore, the contribution of drinking water to the TDFI of 25%, as found in the present study using model simulations, is rather coherent with the values obtained in the aforementioned studies.

The results obtained here suggest that the difference in exposure levels between children from Quebec and Ontario, brought out by the biomonitoring data, cannot be solely explained by the fluoridated drinking water at 0.7 mg/L. Furthermore, it can be deduced from studying Figure 5 that the difference between the two provinces also appears to result from dietary exposure. Since water is used in the preparation of many foods and beverages, it is to be expected that the concentration of fluoride in drinking water will affect the concentration found in the diet, which can explain the difference between both provinces. Taking into account the halo effect, it would be expected that in Quebec, where only 2.5% of the population has access to fluoridated water, fluoride would still be found in foods imported from Ontario or the United States. For example, it would be found where fluoridation is more prevalent. This effect indicates that foods produced in a fluoridated city will contain a higher fluoride concentration than those produced in a non-fluoridated city where it could be distributed and consumed, which increases the fluoride exposure of non-fluoridated areas’ residents and vice-versa [65]. Conversely, modeled dietary intakes do not seem to contribute significantly to the total intake estimated from biomonitoring data in Quebec, according to Figure 5. However, this assertion appears rather uncertain given that modeled data on fluoride levels in foods were taken from a 1969 Nutrition Canada study [11] and may have changed since then based on the population’s dietary habits.

The results also suggest that, at age 4, intake from fluoride toothpaste could be sufficient to reach the suggested optimal intake in the specific population of the present study while, at 8 years of age, the contribution from other sources would also be necessary (Figure 5). However, Ontario’s modeled intake exceeds the suggested optimal intake and, therefore, it is possible that only a fraction of the contribution from the other sources than fluoride toothpaste (drinking water and food) may be sufficient. These fractions are to be determined. This observation is important given that, although fluoride exposure prevents tooth decay, it may trigger adverse health effects particularly fluorosis if the exposure is too high. Setting a drinking water fluoride concentration limit, therefore, requires an ideal compromise between risk and benefits from a dental health perspective [66]. Still, by applying the bioavailability factors previously mentioned to tolerable daily intakes (TDI) recommended by Health Canada (0.1 mg/kg/day) [3,11,15] and, more recently, Australia and New Zealand (0.20 mg/kg/day) [67], it is possible to compute absorbed TDI values determined to be 0.08 mg/kg/day by Canadian public health authorities and 0.16 mg/kg/day by those from Australia/New Zealand. The CHMS biomonitoring data-derived exposure dose for the mean and 95th percentile of Quebec and Ontario can, therefore, be compared to these absorbed TDIs. This allowed us to evaluate the risk of exceeding TDIs when drinking water contains fluoride at concentrations targeted with the aim of reaching a suggested optimal dose against tooth decay.

When comparing those absorbed TDIs with absorbed doses calculated from CHMS data (see Table 4), it can be noted that, on average, the fluoride intake in Quebec and Ontario is lower than the Canadian TDI. However, at age 4, the 95th percentile in Quebec has an intake greater than the Canadian TDI but lower than the Australia/New Zealand TDI. In Ontario, the 95th percentile exceeds the Canadian TDI at ages 4 and 8 and exceeds the Australia/New Zealand TDI at age 4. Conversely, in a context where water is generally not fluoridated as in Quebec, the TDI of Australia/New Zealand is not exceeded. Likewise in Ontario, where most of the population has access to fluoridated water at a level of 0.7 mg/L, children in the 95th percentile may have an intake greater than the TDI of Australia/New Zealand at age 4 and, therefore, presents a potentially increased risk of severe, otherwise rare, dental fluorosis. Additionally, this TDI is twice as permissible as the Canadian one. Yet, while both TDIs are determined in order to be protective of any adverse health effect including fluorosis, it is noteworthy to recall that any TDI does not necessarily correspond to an exposure over which the prevalence of adverse health effects automatically increases, but rather an exposure below which any effect is likely to occur based on the available weight of evidence. Therefore, we consider whether a fraction of the population exceeding a given TDI should be thought of as a safeguard rather than an absolute threshold. Since the dose is expressed in mg/kg, the fluoride intake would have to increase in proportion to body weight in the long-term to remain at 0.2 mg/kg/day and, therefore, increase risks of adverse effects. Increased monitoring using tools such as the urinary fluoride/creatinine ratio (UF/Cr) of 1.69 mg/g suggested by Zohoori and Maguire [68] in order to detect an excessively high fluoride intake well before the onset of dental fluorosis, could be an alternative for amending the water fluoridation guideline.

This study has some limitations that must be acknowledged. In the first place, the PBPK models used are built based on an original one that has seldom, if ever, been used nor validated since its publication. Therefore, this limits the assessment of its validity. However, the validation process realized herein shows that, when corrected appropriately, it can reproduce experimental data rather well (Figure 4). The models are deterministic and represent theoretical average exposure levels in the two ages considered. They, therefore, do not consider the physiological variations in the individuals of the same age group nor of different ages. Neither did they account for the variations in fluoride concentrations in the different exposure sources contrary to the population biomonitoring data used. This may have contributed in some cases to the lack of agreement between modeled and measured urinary fluoride values. In this line, a systematic overestimation by a factor of about 2.5 of the modeled urinary concentration and, therefore, an underestimation of the associated fluoride intake was observed. The model parameters attributable to such overestimations are revealed by the sensitivity analysis (Figure 6).

First, the bone clearance of the model, related to the bone volume in the model, which exhibits a negative AUC-based sensitivity index, may be too low. As a result, the plasma concentration is overestimated with correspondingly overestimated modeled urine concentration. Since bone clearance was calculated with a two-point line assuming that the relationship was linear (see Methods), it is also possible that the values used are not accurate enough. In addition, the model’s overestimation of urinary fluoride could be explained by an excessive modeled renal clearance, which also exhibits a negative AUC-based sensitivity index and a positive Aexc-based one. However, according to the values found in the literature, this does not appear to be the case [8]. Lastly, the absorption fraction appears to be the main contributor to the overestimation. Direct infusion into the liver was considered herein rather than via an intermediate gastrointestinal compartment from which part of the ingested fluoride is absorbed following a rate dictated by an oral absorption constant. The highly model-sensitive correspondent absorption fraction modeled herein (Figure 6) is probably too high as a result. Excessive modeled bioavailability for toothpaste is also a valid explanation. Bioavailability studies are often performed on an empty stomach while tooth brushing is recommended after eating a meal. The ingestion of toothpaste is more likely to happen in the presence of food, which could decrease its true bioavailability. This is supported by a lower modeled Aexc for toothpaste exposure alone than what CHMS data suggests (Figure 5). Unfortunately, the lack of availability of a relevant oral absorption constant and toothpaste bioavailability for different states of stomach emptiness have precluded correcting the model in any other way than applying an empirical modification of the model by a factor of 0.43, which did, however, correct the initial systematic error (Figure 4).

Another limitation related to the fluoride intake may come from dental care or dental hygiene measures other than the use of fluoride toothpaste. Furthermore, this intake was not taken into account in the model due to a lack of data precise enough to be used in the model. However, because professional dental care is provided only a few times each year, it does not appear to be a significant source of long-term exposure, which, in principle, is what is reflected in CHMS biomonitoring data. For other personal fluoride products such as mouthwash, these are not recommended for children under 12 and, therefore, should not affect the estimates in this work. The lack of information on water fluoride levels associated with CHMS biomonitoring data is a source of uncertainty as well.

Two final sources of uncertainties in this study that preclude drawing firm conclusions include the low statistical power of the provincial biomonitoring data for the age groups corresponding to the typical individuals whose exposure was modeled as well as the fact that the child data used to validate the model did not come from Canadian studies despite Canadian biomonitoring data being used in this work. Therefore, since this is the first study of its kind, it needs to be replicated in future studies to validate the findings. In this regard, obtaining more robust data on fluoride levels in foods and their bioavailability in foods for cities with variable levels of fluoride content in the drinking water would improve our assessment particularly with respect to the halo effect of drinking water on food. Probabilistic modeling should be considered, e.g., via Monte Carlo simulations, in order to address the limitation issue related to the inter-individual variability of the various fluoride intakes and of the physiological determinants of its kinetics in children.

## 5. Conclusions

According to the present model, the currently recommended absorbed fluoride dose of 0.04 mg/kg/day to prevent tooth decay is not attained in Quebec children where drinking water fluoridation is sparse while it is surpassed by children in Ontario where such fluoridation is extended. Since this study is the first of its kind regarding the use of biomonitoring data under a PBPK modeling approach in order to compare fluoride intake in children from two regions differing in their access to intentionally fluoridated water, further research needs to be undertaken in order to confirm these results. In addition, these results are an incentive to further explore the multiple sources of fluoride intake since they suggest that a new balance between them are sought, which is in accordance with the physiological differences that influence fluoride metabolism in each age group. This is important from a public health perspective since the aim is to maximize the number of individuals capable of achieving a daily fluoride intake that provides appropriate outcomes in terms of caries prevention and minimizing the risk of fluorosis.

## Figures and Tables

**Table 1:** Fluoride intake and urinary fluoride measured in the studies were selected to validate the model.

Age (years)	Children (*n*)	Intake (mg/day)				AuF-24 ^1^ (mg/day)	Country
		Diet	Toothpaste	Water	Supplement		
4	31	0.560	0.706	0.042	-	0.3682	Venezuela [37]
4	20	0.533	0.254	0.231	-	0.358	Chile [38]
5	61	0.151	0.608	0.407	-	0.3705	UK [39]
5	11	0.092	0.274	0.111	0.455 ^2^	0.476	Germany [40]
7	21	0.187	0.606	0.154	-	0.297	UK [41]
7	12	0.229	1.130	0.349	-	0.393	UK [41]

^1^ Amount of urinary fluoride excreted in 24 h. ^2^ Fully absorbed fluoride tablets.

**Table 2:** Age-specific fixed parameter values needed to model fluoride exposure.

Age (year)	Weight (kg)	Height (cm)	Sources of Fluorides				
			Toothpaste (µg/kg/day)	Diet (µg/kg/day)	Soil (µg/kg/day)	Air (µg/kg/day)	Water (L/day)
4	16	103	40	21	1.19	0.01	0.442
8	25	127	30	17.5	0.21	0.01	0.56

**Table 3:** Urinary fluoride concentrations (mg/L) modeled in a 4-year-old and an 8-year-old child for different exposure scenarios compared with Province-specific CHMS biomonitoring data.

Province	Scenario	Age-Specific Results					
		4 year-old Child			8 year-old Child		
		Model	CHMS	Ratio ^1^	Model	CHMS	Ratio ^1^
Ontario	1. (Fluoridation of drinking water at 0.7 mg/L)	0.846	0.83	1.02	0.73	0.67	1.09
Quebec	2. (Fluoridation at 0.06 mg/L with dietary intake)	0.708	0.39	1.81	0.605	0.34	1.78
Quebec–modified	3. (Fluoridation at 0.06 mg/L without dietary intake)	0.574		1.47	0.48		1.41

^1^ Ratio of the modeled urinary concentration over the CHMS bio-monitored urinary concentration.

**Table 4:** Absorbed fluoride doses (mg/kg/d) modeled with CHMS biomonitoring data from Quebec and Ontario.

Age	Quebec, Geometric Mean (95th Percentile)	Ontario, Geometric Mean (95th Percentile)
4	0.03 (0.13)	0.06 (0.17)
8	0.02 (0.05)	0.05 (0.12)

## Appendix A

**Table A1:** Absorbed doses calculated from the McClure [1] data on intakes from water and diet ^1^.

Age (year)	Weight (kg)	Water (mL/day)	Diet (mg/day)	Total (mg/day)	Intake (mg/kg/day)	Absorbed Dose (mg/kg/day) ^1^
1 to 3	8 à 16	0.39–0.56	0.027–0.265	0.417–0.825	0.026–0.103 (0.065)	0.036–0.058 (0.047)
4 to 6	13 à 24	0.52–0.745	0.036–0.360	0.556–1.105	0.023–0.085 (0.054)	0.030–0.049 (0.040)
7 to 9	16 à 35	0.65–0.93	0.045–0.450	0.695–1.380	0.020–0.086 (0.053)	0.029–0.048 (0.039)
10 to 12	25 à 54	0.81–1.66	0.056–0.560	0.866–1.725	0.016–0.069 (0.043)	0.023–0.037 (0.031)

^1^ Absorbed optimal doses were calculated by multiplying mean water and diet intake by their respective bioavailability factors of 83% and 40% and dividing that total absorbed dose by the mean weight for that age group.
